# Adjuvant treatment with Xiaoqinglong formula for bronchial asthma: Protocol of systematic review and meta-analysis

**DOI:** 10.1097/MD.0000000000017053

**Published:** 2019-08-30

**Authors:** Long Wang, Xiuli Zheng, Yi Hui, Baojia Wang, Yu Yang, Xianrong Feng, Tianyao Zhang, Li Ma, Xiaobo Zhang

**Affiliations:** a Chengdu University of Traditional Chinese Medicine; b Hospital of Chengdu University of Traditional Chinese Medicine, Chengdu, Sichuan; c Shaanxi University of Traditional Chinese Medicine, Xianyang, Shaanxi, China.

**Keywords:** bronchial asthma, protocol, systematic review, Xiaoqinglong decoction

## Abstract

**Background:**

Bronchial asthma is one of the most common chronic diseases in the world and has become a serious public health problem. Combination therapy has become the first choice for clinical treatment of bronchial asthma. In addition to the combined use of routine medication, traditional Chinese medicine as an adjuvant therapy is also considered. Xiaoqinglong Decoction (XQLD) is an effective prescription of traditional Chinese medicine in treating asthma, and there are more and more clinical reports about its combination with western medicine in treating asthma. Therefore, we designed this study protocol to evaluate the adjuvant role of XQLD in the treatment of bronchial asthma.

**Method:**

The following electronic databases will be systematically searched from inception to April 2019: PubMed, EMBASE database, Cochrane Library, China National Knowledge Infrastructure (CNKI), Wan Fang, Chinese Scientific Journals Database (VIP), and China Biology Medicine disc, (CBM). And the following primary outcomes will be tested, including effective rate (ER), pulmonary function (FEV1, PEF, FEV1/FVC), adverse reactions (AR). RevMan5 software will be used for literature quality evaluation and stata14.0 software will be used for data synthesis and analysis.

**Result:**

To evaluate the efficacy and safety of Xiaoqinglong decoction in combination therapy by observing the outcomes of efficacy, adverse reactions and pulmonary function.

**Conclusion:**

This study protocol will be used to evaluate the efficacy and safety of XQLD in combination with conventional drugs in the treatment of bronchial asthma, as well as the adjuvant role of XQLD in combination.

**PROSPERO registration number:**

CRD42019133549

## 1 Introduction

Bronchial asthma is a chronic airway inflammatory disease involving a variety of inflammatory cells and cytokines.^[1]^ This disease is mostly caused by extensive and changeable airflow obstruction.^[2]^ It is a clinically refractory disease characterized by severe asthma, inability to lie down, shortness of breath in the nose and chest tightness. At present, there are about 300 million asthmatic patients in the world, and about 30 million of asthmatic patients in China. Asthmatic patients are increasing year by year because of its slow onset and difficulty in rapid recovery.^[3]^ In recent years, with the aggravation of climate and environmental pollution, the incidence of asthma in the world is increasing quickly, so how to effectively prevent and treat bronchial asthma is an urgent problem to be solved. The understanding of asthma in traditional Chinese medicine is based on the theory of traditional Chinese medicine, which has accumulated rich experience in the treatment of asthma. XQLD is an effective prescription of traditional Chinese medicine for asthma, which has been widely used in clinical in China.^[4]^ Experimental studies found that XQLD may play a role in reducing inflammation and alleviating asthma by regulating TSLP signaling pathway.^[5]^ Pharmacological studies also confirmed that ephedra, Cinnamon Twig and licorice in XQLD have anti-inflammatory and anti-allergic effects. Combination medication is the most effective way to treat bronchial asthma. Therefore, in China, clinicians often use XQLD to treat asthma on the basis of routine western medicine treatment, and have achieved good therapeutic effect. For example, 1 study found that inhaled fluticasone combined with XQLD had better effects on pulmonary function and serum interleukin-16 level in asthmatic patients than inhaled fluticasone alone.^[6]^ In clinic, there are more and more reports about XQLD combined with routine western medicine in the treatment of bronchial asthma, but the effectiveness and safety of this combination is lack of systematic evaluation, so we design this protocol to systematically evaluate its effectiveness and safety, as well as its adjuvant role in the combined treatment.

## 2 Method

### 2.1 Inclusion criteria

#### 2.1.1 Types of study

According to Cochrane Collaboration's RCT criteria, all references to the words “random sequence ” in the article are regarded as RCTs, regardless of whether they are single-blind, double-blind or non-blind. Languages are limited to Chinese and English.

#### 2.1.2 Study participants:

Exclusion of congenital heart disease, lung cancer, bronchiectasis, pneumonia, liver, and kidney impairment and other systemic diseases; diagnostic criteria: Guidelines for the Prevention and Treatment of Bronchial Asthma Revised by Respiratory Society of Chinese Medical Association (2008);^[7]^ And regardless of age, severity, and duration of the disease. Follow-up period is longer than 6 months.

#### 2.1.3 Intervention

Interventions in the experimental group are XQLD combined with conventional western medicine. XQLD must contain the original drug composition regardless of the dosage form (tablet, mixture, decoction). The interventions in the control group are routine drug therapy in western medicine, including bronchodilator, expectorant, glucocorticoid, β 2 receptor agonist, sustained release theophylline and so on.

#### 2.1.4 Outcomes

Primary outcomes:

(1) Total clinical efficacy rate: clinical effectiveness includes clinical control, effectiveness and remarkable result. After treatment, the number of incidents was counted to calculate the efficiency rate.
(2) Lung function: using forced expiratory volume in1 second (FEV1), forced vital capacity (FVC), and peak expiratory flow (PEF) as indicators of lung function.
(3) Adverse reactions: after the start of treatment, the main symptoms of adverse reactions will be recorded, and the number of patients with adverse reactions will be counted.

Additional outcomes:

(1) Recurrence rate: during the follow-up period, record the recurrence of patients by telephone.
(2) Serum Ig E was measured by ELISA kit before and after treatment in 2 groups.

### 2.2 search strategy

This meta-analysis will be conducted according to the recommendations of the Preferred Reporting Items for Systematic Reviews and Meta-analyses Statement.^[8]^ The following electronic databases will be systematically searched from inception to April 2019: PubMed, EMBASE database, Cochrane Library, China National Knowledge Infrastructure (CNKI), Wan Fang, Chinese Scientific Journals Database (VIP), and China Biology Medicine (CBM). In addition, we also manually search additional relevant studies, using references from systematic reviews published previously. The following key words or phrases and their abbreviations or derivatives are utilized singly or in combination: “Chinese herbal medicine” or “Chinese medicine” or “traditional Chinese medicine” or “TCM” or “Xiaoqinglong” or “Xiaoqinglong decoction” or “Xiaoqinglong tang” or “XQLD” or “asthma” or “asthmas” or “bronchial Asthma” or “asthma, bronchia”.

### 2.3 Studies Selection

**Figure 1:**
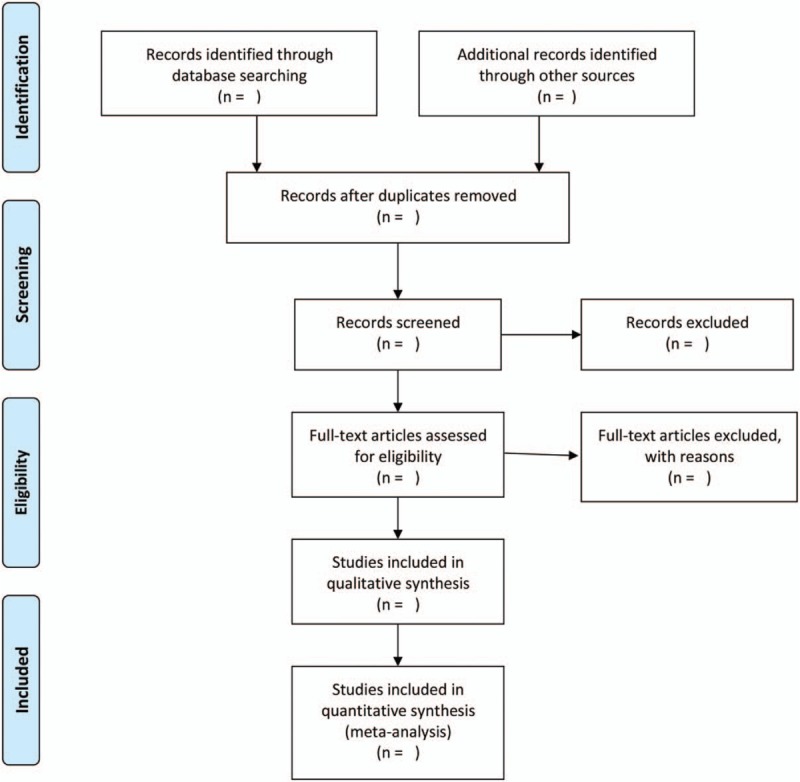
Preferred reporting items for systematic reviews and meta-analyses flow chart of study selection process.

Two reviewers (BJ Wang and XR Feng) independently screen the titles and abstracts of searching results against prespecified inclusion criteria to identify potential relevance. Disagreements are resolved by consensus. All articles included are judged by the third reviewer (Yu Yang). The whole selection process will be presented in a PRISMA flow diagram (Fig. 1).

### 2.4 Data extraction

Two reviewers (BJ Wang and XR Feng) will search these databases and independently evaluate all the eligible articles for inclusion. Disagreements will be resolved by consensus. If they do not reach an agreement on a given article, the third reviewer will make the final decision. An excel data extraction form will be devised and piloted in selected studies. Quantitative data for meta-analysis will be extracted on a separate extraction sheet.

### 2.5 Risk of bias (quality) assessment

The methodological quality of the included trials will be evaluated using the Cochrane Handbook for Systematic Reviews of Interventions. It mainly includes the following: selection bias (random sequence generation and allocation concealment), performance bias (blinding of participants and personnel), detection bias (blinding of outcome assessment), attrition bias (incomplete outcome data), reporting bias (selective reporting), and other sources of bias. Quality of each item will be divided into low/unclear/high risk of bias. Any disagreements will be analyzed by the third reviewer (Yu Yang).

### 2.6 Strategy for data synthesis

Stata14.0 will be used for this statistical analysis. Risk ratio (RR) will be used for dichotomous outcomes and weighted mean difference (WMD) will be adopted for continuous outcomes. Homogeneity of risk estimates between studies is assessed using the *I*^2^ statistic and the Cochran Q statistic. Data will be analyzed with a fixed-effect model if no statistical heterogeneity is indicated (*I*^2^ < 50% or *P* > .10); otherwise if statistical heterogeneity is indicated (*I*^2^ > 50% or *P* < .10), pooled effect sizes are calculated by the random-effects model. The confidence interval (CI) will be established at 95%. Publication bias will be explored by funnel plot analysis, and quantitative test of publication bias by Begger method.

### 2.7 Analysis of subgroups or subsets

Two pre-specified subgroup analyses will be carried out

(1) age: adults (≥18) and children (<18).
(2) Clinical stage: acute episode and chronic persistence.

### 2.8 Sensitivity analysis

If the heterogeneity between studies remains high after subgroup analysis, then sensitive analysis is performed. After excluding a low-quality study, meta-analysis is conducted again to compare the new merger results with those before. If there is no significant change between the 2 results, the sensitivity would be low; On the contrary, if the excluded merger results differ greatly from the original merger results, or even get the opposite conclusion, then the sensitivity is high.

### 2.9 Ethics and dissemination

This review is going to be shed in peer-reviewed journals. Our research is a systematic review, which does not contain personal information of patients. Therefore, informed consent and ethical permission are not required for our research.

## 3 Discussion

Bronchial asthma is the most common chronic respiratory disease. In recent years, with the aggravation of environmental pollution, the incidence of asthma has increased year by year. Its pathogenesis is complicated, and is not completely clear at present. About treatment of asthma, there is not any radical cure, and only can be controlled by drugs.^[9]^ The treatment of acute episode focuses on anti-inflammation and anti-infection to relieve symptoms as soon as possible, so high doses of glucocorticoids should be used.^[10]^ And glucocorticoids should be inhaled regularly for a long time to inhibit airway inflammation, decrease the frequency of acute episode and improve irreversible damage of pulmonary function with asthma chronicity-persistent period. After standardized treatment, most patients can achieve good control effect, but there are still a few patients with a long course of disease, poor control effect and recurrent attacks. Therefore, clinicians should also look for other therapeutic approaches while implementing prevention and treatment guidelines. In China, TCM therapy has been widely used in clinical practice. Chinese medicine believes that asthma occurs because of the obstruction of “Fei Qi”, which cannot be dredged, leading to asthma attack, cough, and other manifestations.^[11,12]^ XQLD is a common prescription of traditional Chinese medicine for treating bronchial asthma. It can dredge flow of “fei qi” and improve airflow restriction. And its therapeutic effect has been affirmed in clinical. We draft this protocol in order to observe the efficacy and safety of conventional drug therapy combined with XQLD, and its auxiliary role in combination. We set up 2 subgroups in advance for analysis. The first one was divided into children and adults, considering that the incidence of adult asthma is more unstable, the recurrence rate is higher and the remission rate is lower than that of childhood asthma.^[13]^ Another subgroup was classified according to the clinical stage of asthma. On acute attack of asthma, treatment should focus on rapid relief of symptoms. Therefore, large doses of glucocorticoids and bronchodilators should be used. And the duration of asthma should be maintained with continuous low doses of glucocorticoids, antibiotics, and leukotriene antagonists. Therefore, there are obvious differences in dosage and choice of drugs. Effective rate and pulmonary function are the main outcome indicators. Effective rate can directly reflect the therapeutic effect. Pulmonary function was evaluated by FEV1, PEF, and FEV1/FVC, which is the main reference index for diagnosis and observation of the therapeutic effect.^[14]^ This protocol can ensure the normal implementation of this research through scientific design, standardized methods and rational personnel arrangement.

## Author contributions

**Conceptualization:** Long Wang, Tianyao Zhang, Li Ma.

**Data curation:** Baojia Wang, Xianrong Feng.

**Formal analysis:** Xiuli Zheng.

**Funding acquisition:** Long Wang, Yi Hui.

**Investigation:** Li Ma, Xiaobo Zhang.

**Methodology:** Tianyao Zhang.

**Project administration:** Xiuli Zheng, Yu Yang.

**Resources:** Yi Hui.

**Software:** Long Wang, Baojia Wang, Tianyao Zhang, Li Ma, Xiaobo Zhang.

**Supervision:** Yu Yang.

**Validation:** Xianrong Feng.

**Writing – original draft:** Long Wang.

**Writing – review & editing:** Yu Yang.
